# Analysis of the correlation between heart rate variability and palpitation symptoms in female patients with long COVID

**DOI:** 10.3389/fcvm.2023.1273156

**Published:** 2023-11-17

**Authors:** Yu Jiang, Yan Cheng, Jingwen Xiao, Yicheng Wang, Geng Chen, Yan Zhang

**Affiliations:** ^1^ Department of Cardiovascular Medicine, Fuzhou First Hospital Affiliated with Fujian Medical University, Fuzhou, China; ^2^ The Third Clinical Medical College, Fujian Medical University, Fuzhou, China; ^3^ Cardiovascular Disease Research Institute of Fuzhou City, Fuzhou, China; ^4^ Department of Nursing, Fuzhou First Hospital Affiliated with Fujian Medical University, Fuzhou, China

**Keywords:** long COVID, palpitations, heart rate variability, autonomic dysfunction, autonomic nervous system

## Abstract

**Objectives:**

To analyze the correlation between heart rate variability (HRV) and palpitation symptoms in female patients with long COVID.

**Methods:**

A total of 272 female healthcare workers who were infected with SARS-CoV-2 for the first time in December 2022 at Fuzhou First Hospital affiliated with Fujian Medical University, were selected as study subjects. These subjects were divided into three groups based on their symptoms: a group with palpitations (70 cases), a group without palpitations but with other symptoms (124 cases), and a group consisting of asymptomatic cases (78 cases). The study compared the general information, COMPASS-31 scores, quality of life scores, and HRV parameters among the three groups. Furthermore, it analyzed the factors influencing palpitation symptoms in female patients with long COVID.

**Results:**

Compared to the other two groups, the HRV parameters SDNN, HRVIndex, LF, and TP were significantly reduced in the group with palpitations (*p *< 0.05). Multivariate analysis revealed that HRVIndex (*p *= 0.016; *OR*: 0.966, *95% CI*: 0.940∼0.994) had a significant impact on palpitation symptoms in female patients with long COVID.

**Conclusions:**

The symptoms of palpitations in female patients with long COVID were found to be related to HRV parameters. Autonomic dysfunction may be connected to the occurrence of palpitation symptoms in long COVID.

## Introduction

Long COVID, also known as ‘post-acute sequelae of COVID-19' or ‘post-COVID-19', is a multisystemic disorder characterized by severe symptoms that occur following infection with the severe acute respiratory syndrome coronavirus 2 (SARS-CoV-2). According to the World Health Organization (WHO), the post-COVID-19 state typically occurs 3 months after the onset of COVID-19, and the symptoms last for at least 2 months (1). These symptoms, which cannot be explained by any other diagnosis, include palpitations, fatigue, shortness of breath, chest pain, headache, memory loss, and various other manifestations.

Palpitations are sensations of heart activity, often described as a fluttering, running, or jumping sensation. Palpitations can occur in people without heart disease or can be caused by a life-threatening heart condition (2). Clinical investigations have shown that palpitations occur in approximately 10%–50% of patients weeks to months after SARS-CoV-2 infection (3, 4), with a higher prevalence among female patients (5, 6). Palpitations following SARS-CoV-2 infection may be closely associated with autonomic dysfunction (7, 8).

Autonomic dysfunction originates from disorders that directly affect the autonomic nerves and also reflects changes in autonomic function secondary to cardiac or other disorders (9). Heart rate variability (HRV) is one of the strongest predictors of autonomic function. HRV is a non-invasive method that provides some indicators for evaluating the regulation of the autonomic nervous system on the sinus node (10). HRV has been shown to be useful in the detection and evaluation of palpitations in patients with benign paroxysmal positional vertigo and fibromyalgia (11, 12). However, there is limited research on the correlation between HRV and palpitations in individuals with long COVID (13). This study aims to analyze the relationship between palpitation symptoms and HRV parameters in female patients with long COVID to gain a better understanding of the autonomic mechanism behind these symptoms. The findings of this study will provide valuable data for further research on changes in autonomic function in patients with long COVID.

## Methods

### Study design and ethics

**Figure 1:**
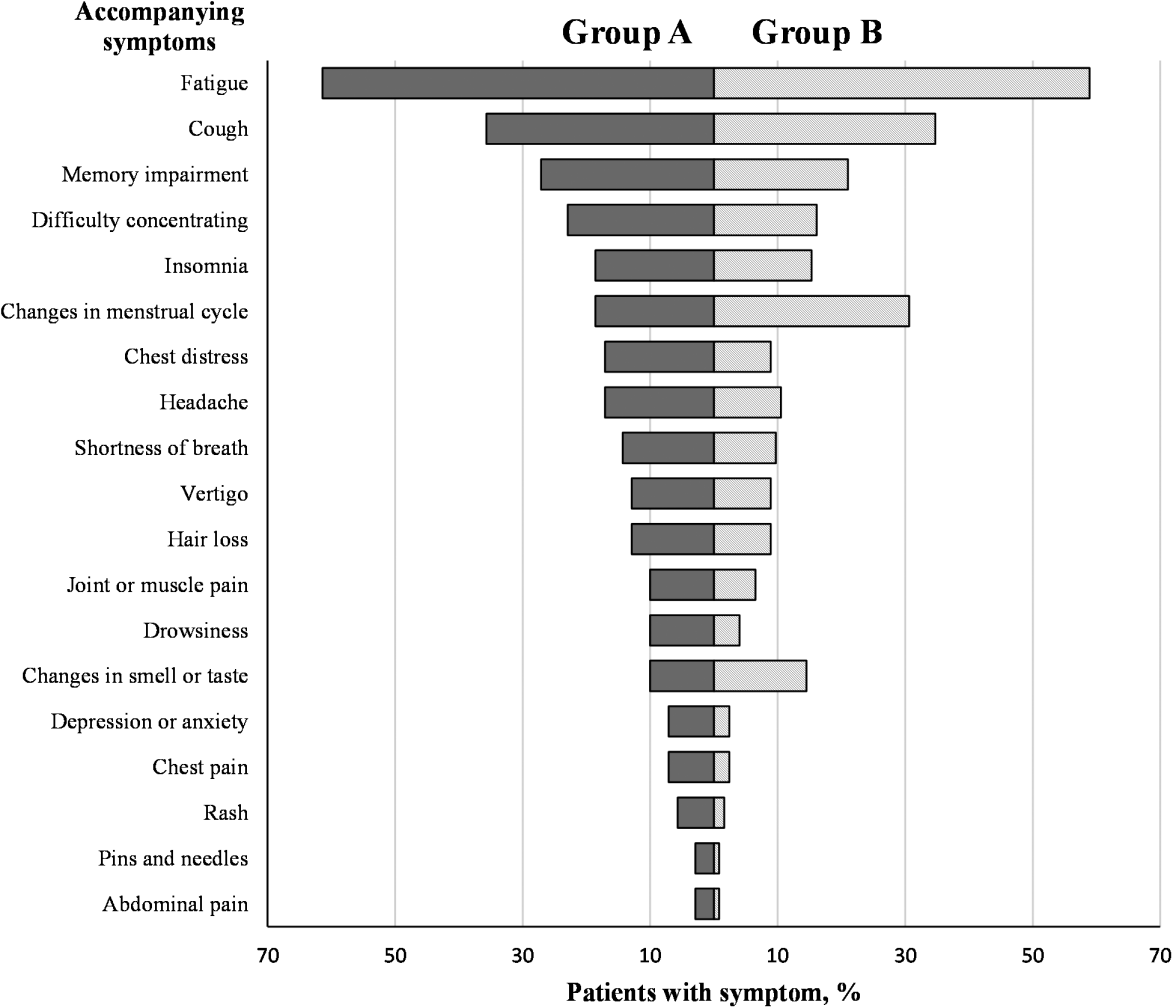
Comparison of accompanying symptoms between group A and group B.

A total of 701 cases of female healthcare workers who were initially infected with the SARS-CoV-2 virus in Fuzhou First Hospital affiliated with Fujian Medical University in December 2022 were included in this study. After applying the inclusion and exclusion criteria, 272 cases were selected as the study subjects. Patients completed the long COVID Symptoms Questionnaire on the basis of the COVID-19 Yorkshire Rehabilitation Scale (C19-YRS) (14, 15) and were categorized as having or not having palpitations using an individual item from the Menopausal Symptoms Scale (16). Based on the results of these scales, the patients were divided into three groups: Group A (70 cases) consisted of patients with symptoms of palpitations, with or without other symptoms associated with long COVID (e.g., fatigue, cough, insomnia, headache, chest distress, shortness of breath, etc., as shown in Figure 1), Group B (124 cases) included patients who did not have palpitations but exhibited other symptoms such as fatigue, cough, insomnia, headache, chest distress, shortness of breath, etc., and Group C (78 cases) comprised asymptomatic cases with a history of COVID-19. In line with the definition of long COVID, patients in groups A and B were classified as long COVID patients, whereas patients in group C did not develop into long COVID patients.

The inclusion criteria for this study were as follows: (a) individuals who were all first infected with SARS-CoV-2 in December 2022; (b) employees who underwent health checkups in 2023 and showed no diseases in vital organs such as the heart, lungs, brain, liver, kidneys, and spleen; (c) employees whose resting twelve-lead electrocardiogram showed sinus rhythm at the health checkups; (d) The participants provided written informed consent before enrolling in the study and all research procedures were carried out in accordance with the Declaration of Helsinki.

The following exclusion criteria were applied: (a) individuals with a previous history of arrhythmias such as atrial fibrillation, atrial flutter, or pacemaker implantation; (b) individuals with a previous history of diabetes mellitus, bronchial asthma, allergic rhinitis, or gastritis; (c) individuals with a previous history of malignant tumor or other serious consumptive diseases; (d) individuals with comorbidity of serious physical diseases and mental and psychological disorders; (e) individuals with a history of smoking or alcoholism; (f) individuals with a recent history of trauma; (g) individuals with recent consumption of beta-blockers, anti-psychotics, or other drugs that affect autonomic function.

This study was approved by the Ethics Committee of Fuzhou First Hospital affiliated with Fujian Medical University (approval number 202308002).

### Collection of general information

The study collected general data from the patients, including age, height, weight, night shift situation, regular exercise habits (moderate-intensity exercise lasting 30–45 min or more per session, ≥3 days per week), and the results of the questionnaire on the symptoms of long COVID.

### Palpitation

Symptoms of palpitations were assessed on a four-point Likert scale in the fourth item of the Menopausal Symptoms Scale: none (never, 0 points), mild (rarely, 1 point), moderate (sometimes, 2 points), or severe (very often, 3 points). Based on validated and utilized studies, “absent” or “mild” was defined as not having palpitations, and “moderate” or “severe “ was defined as having palpitations (16).

### Heart rate variability detection

The 5 min HRV parameters were collected using an ECG-512A automatic ECG analyzer (Turui Technology Co., Nanjing, China; the sampling frequency of the ECG recording was 400 Hz). HRV testing was performed in April 2023 in the cardiac function room of Fuzhou First Hospital affiliated with Fujian Medical University. The testing setting was kept quiet and comfortable with the room temperature at 24–26°C. HRV testing in female participants was scheduled within one week of the end of menstruation. Participants were instructed to maintain adequate sleep 24 h before the test, avoid strenuous exercise and emotional fluctuations, and refrain from consuming coffee, milk tea, and other foods that may affect HRV detection. Before the test, participants were required to rest quietly for 30 min. During the test, the participants were kept in the supine position without any further challenge.

The multichannel electronic data recording system's ECG analyzer software (Turui Technology Co., Nanjing, China) was utilized for the analysis of HRV, this system transmits and analyzes the gathered ECG data, while also reviewing it through the expertise of two ECG lab physicians. Methods for assessing HRV include time-domain analysis and frequency-domain analysis. The time-domain parameters assessed include: (a) Mean R-R, which denotes the mean of R-R intervals; (b) SDNN, which represents the standard deviation of all R-R intervals; (c) rMSSD, which indicates the root mean square of the difference between adjacent R-R intervals throughout the entire process; (d) PNN50, which suggests the percentage of the differences between adjacent normal R-R intervals exceeding 50 ms; and (e) HRV delta index (HRVIndex), calculated as the total number of R-R intervals divided by the height of the histogram of R-R intervals. The frequency-domain parameters assessed are: (a) Total Power (TP); (b) Very Low-Frequency Power (VLF); (c) Low-Frequency Power (LF); (d) High-Frequency Power (HF) and (e) LF/HF (17). The time-domain parameters and frequency-domain parameters have well-defined meanings and established theories, and they have been extensively utilized in clinical studies (9).

### COMPASS-31

The Composite Autonomic Symptom Score 31 (COMPASS-31) is a useful tool for evaluating a patient's autonomic function. The questionnaires were administered and completed by professionally trained medical personnel. The COMPASS-31 total score ranges from 0 to 100. A higher score on the COMPASS-31 indicates the presence of more severe autonomic symptoms (18). COMPASS-31 can be used as a sensitive tool to detect the possibility of autonomic dysfunction in long COVID (19).

### Quality of life scores

Quality of life was assessed using the EuroQol visual analog scale before and 3 months after SARS-CoV-2 infection. Scores ranging from 0 to 100 were assigned, with a difference of 10 points indicating a decline in quality of life (20).

### Statistical analysis

Statistical analysis was performed using SPSS 26.0. Normally distributed measures were presented as mean ± standard deviation (x ± s), while skewed measures were presented as median (quartiles) [M (P25, P75)]. The Mann–Whitney *U*-test was used to compare the two groups, and the Kruskal-Wallis test was used for multiple comparisons. Count data were expressed as the number of cases and percentage, and the chi-square test was used for group comparisons.

The binary logistic regression model was utilized to analyze the influencing factors. Eigenvalues and condition indices were used to evaluate multicollinearity. The logarithmic transformation of each parameter was employed to assess linearity. Initially, four meaningful parameters, namely SDNN, HRVIndex, LF, and TP, were identified by comparing between-group variability. However, TP was excluded due to its high correlation with SDNN and the presence of multicollinearity. Finally, multivariate analysis was conducted using long COVID palpitation symptoms as the dependent variable (with palpitation symptoms = 1, without palpitation symptoms = 2), and SDNN, HRVIndex, and LF as independent variables. Based on the EPV (event for each variable) method, the result indicated that a total sample size of 115 cases (EPV of 10) was required. The sample size of this study meets the requirement.

Statistical significance was defined as *p *< 0.05.

## Results

A total of 272 subjects were included in the study. Among these subjects, Group A included 70 patients with palpitations with or without other symptoms, Group B included 124 patients without palpitations but with other symptoms, and Group C included 78 asymptomatic patients. The main characteristics of the patients in each group are shown in Table 1. There were no significant differences between the three groups in terms of age, BMI, night shift status, and regular exercise habits (*p > *0.05). Additionally, the COMPASS-31 scores were significantly higher in Groups A and B than in Group C, with a higher score in Group A. The proportion of cases with worsened quality of life was significantly higher in Groups A and B than in Group C, with a higher proportion in Group A (*p *< 0.05).

**Table 1:** Comparison of general information, COMPASS-31, quality of life scores, and HRV parameters in groups A, B, and C.

Characteristics	Group A (70 cases)	Group B (124 cases)	Group C (78 cases)	*H*/*x^2^*	*p*
Age (years)	33.00 (28.00,42.25)	31.50 (29.00,37.00)	33.50 (27.00,40.00)	0.775	0.679
BMI (kg/m^2^)	22.86 ± 2.09	22.46 ± 2.09	22.22 ± 1.44	4.920	0.085
Night shift, No. (%)	33 (47.14)	71 (57.26)	32 (41.03)	5.354	0.069
Exercise regularly, No. (%)	15 (21.43)	13 (10.48)	9 (11.54)	4.957	0.084
COMPASS-31	8.00 (5.00,13.00)^a^^,^^b^	6.00 (2.00,9.75)^a^	0.00 (0.00,3.25)	65.314	<0.001
Worsened quality of life, No. (%)	46 (65.71)^a^^,^^b^	51 (41.13)^a^	15 (19.23)	32.910	<0.001
Mean R-R (ms)	706.50 (628.25,776.00)^a^	747.00 (672.50,825.75)^a^	772.00 (702.00,829.50)	23.503	<0.001
SDNN (ms)	51.18 (36.09,83.44) ^a^^,^^b^	62.24 (43.57,107.44)^a^	100.96 (65.81,166.76)	26.555	<0.001
rMSSD (ms)	46.67 (28.44,73.71)^a^	55.03 (36.43,94.13)^a^	100.87 (60.70,160.02)	27.505	<0.001
PNN50 (%)	11.68 (3.11,23.36)	15.15 (5.27,32.26)	13.94 (8.74,21.17)	1.510	0.470
HRVIndex	40.52 (34.83,49.65)^a^^,^^b^	47.11 (40.51,56.36)^a^	44.57 (40.12,52.61)	10.037	0.007
VLF (ms^2^)	437.77 (224.30,1,222.95)^a^	736.52 (268.95,2,243.50)^a^	1,300.05 (559.40,2,314.30)	16.509	<0.001
LF (ms^2^)	194.09 (80.21,465.06)^a^^,^^b^	391.78 (137.17,1,112.28)^a^	733.50 (397.15,1,523.47)	26.703	<0.001
HF (ms^2^)	316.90 (136.52,794.15)^a^	522.67 (190.61,1,678.79)^a^	950.87 (506.30,3,875.79)	22.554	<0.001
LF/HF	0.65 (0.36,1.26)	0.65 (0.32,1.38)	0.56 (0.28,1.05)	1.420	0.492
TP (ms^2^)	1,083.54 (483.54,3,057.53)^a^^,^^b^	1,859.42 (801.97,6,036.66)^a^	4,161.96 (2,251.09,7,806.22)	21.136	<0.001

H, statistics for the Kruskal–Wallis test (H test); *x^2^*, statistics for chi-square test; BMI, body mass index; SDNN, standard deviation of all R-R intervals; Mean R-R, mean of R-R intervals; rMSSD, square root of the mean squared difference of successive R-R intervals; PNN50, which suggests the percentage of the differences between adjacent normal R-R intervals exceeding 50 ms; HRVIndex, heart rate variability; VLF, very low frequency; LF, low frequency; HF, high frequency; TP, total power.

^a^ Comparison with Group C, *p *< 0.05.

^b^ Comparison with Group B, *p *< 0.05.

There were no significant differences between groups in HRV parameters, i.e., PNN50 and LF/HF (*p > *0.05). The levels of SDNN, HRVIndex, LF, and TP were lower in Group A compared to the other two groups. Both Group A and Group B exhibited lower levels of Mean R-R, SDNN, rMSSD, HRVIndex, VLF, LF, HF, and TP in comparison to Group C. These differences were found to be statistically significant (*p *< 0.05), as shown in Table 1.

In Group A, a higher proportion of symptoms reported were fatigue (61.43%), cough (35.71%), and memory impairment (27.14%). On the other hand, in Group B, a higher proportion of symptoms reported were fatigue (58.87%), cough (34.68%), and changes in the menstrual cycle (30.65%), as shown in Figure 1 and Table 2. The difference in concomitant symptoms between the two groups was not statistically significant (*p *> 0.05), as indicated in Table 2.

**Table 2:** Comparison of accompanying symptoms between group A and group B.

Accompanying symptoms	Group A (70 cases)	Group B (124 cases)	*p*
Fatigue, No. (%)	43 (61.43)	73 (58.87)	0.727
Cough, No. (%)	25 (35.71)	43 (34.68)	0.884
Memory impairment, No. (%)	19 (27.14)	26 (20.97)	0.328
Difficulty concentrating, No. (%)	16 (22.86)	20 (16.13)	0.247
Insomnia, No. (%)	13 (18.57)	19 (15.32)	0.558
Changes in the menstrual cycle, No. (%)	13 (18.57)	38 (30.65)	0.067
Chest distress, No. (%)	12 (17.14)	11 (8.87)	0.087
Headache, No. (%)	12 (17.14)	13 (10.48)	0.184
Shortness of breath, No. (%)	10 (14.29)	12 (9.68)	0.331
Vertigo, No. (%)	9 (12.86)	9 (7.26)	0.197
Hair loss, No. (%)	9 (12.86)	11 (8.87)	0.387
Joint or muscle pain, No. (%)	7 (10.00)	8 (6.45)	0.374
Drowsiness, No. (%)	7 (10.00)	5 (4.03)	0.098
Changes in smell or taste, No. (%)	7 (10.00)	18 (14.52)	0.367
Depression or anxiety, No. (%)	5 (7.14)	3 (3.42)	0.112
Chest pain, No. (%)	5 (7.14)	3 (3.42)	0.112
Rash, No. (%)	4 (5.71)	2 (1.61)	0.113
Pins and needles, No. (%)	2 (2.86)	1 (0.81)	0.266
Abdominal pain, No. (%)	2 (2.86)	1 (0.81)	0.266

Multivariate analysis revealed that HRVIndex (*p *= 0.016; *OR*: 0.966, *95% CI*: 0.940–0.994) had a significant impact on palpitation symptoms in female patients with long COVID, as presented in Table 3.

**Table 3:** Analysis of factors influencing palpitation symptoms in female patients with long COVID.

Characteristics	*β*	*SE*	*WaldX2*	*p*	*OR*	*95% CI*
SDNN	0.001	0.002	0.201	0.654	1.001	0.997–1.005
HRVIndex	−0.034	0.014	5.770	0.016	0.966	0.940–0.994
LF	<0.001	<0.001	<0.001	0.998	1.000	1.000–1.000

*β*, beta coefficient; *SE*, standard error; *WaldX2*, Wald Chi-square; *OR*, odds ratio; *CI*, confidence interval; SDNN, standard deviation of all R-R intervals; HRVIndex, heart rate variability; LF, low frequency.

## Discussion

The main findings of this study indicate that in female patients infected with the SARS-CoV-2 for more than 3 months, several HRV parameters such as Mean R-R, SDNN, rMSSD, HRVIndex, VLF, LF, HF, and TP were found to be lower in symptomatic patients compared to asymptomatic patients. Additionally, it was observed that SDNN, HRVIndex, LF, and TP were significantly lower in long COVID female patients with palpitations. Lastly, HRVIndex was a significant factor for palpitation symptoms in female patients with long COVID.

The rate at which spontaneous action potentials are generated within the sinus node determines the intrinsic heart rate. Activation of *β*-adrenergic receptors on sinus node myocytes by norepinephrine increases heart rate through the sympathetic nervous system, by accelerating the discharge of sinus node action potentials. On the other hand, the parasympathetic nervous system decreases heart rate by releasing acetylcholine, which activates muscarinic M_2_ receptors on sinus node myocytes, resulting in a slowing down of spontaneous action potential firing in the sinus node. Under normal circumstances, sympathetic and vagal functions are balanced. However, when a disorder disrupts the autonomic balance, HRV changes accordingly (21, 22). Among the HRV parameters, SDNN is an important indicator of the overall regulation of the cardiac autonomic nervous system. The rMSSD and PNN50 mainly reflect the functional status of the vagus nerve. HRVIndex represents the overall magnitude of heart rate variability. LF is subject to both sympathetic and vagal modulation, but it has also been suggested that it is primarily influenced by sympathetic nerves. HF primarily reflects vagal modulation and is influenced by respiratory depth. LF/HF quantitatively assesses the equalization of sympathetic and vagal tone. TP reflects the overall activity of the autonomic nervous system. VLF accounts for only a very small fraction of the total power and is primarily influenced by peripheral vasodilation and the renin-angiotensin system (23). Our findings indicate that most HRV parameters were decreased in female patients with long COVID compared with the asymptomatic group, except for PNN50 and LF/HF. Furthermore, SDNN, HRVIndex, LF, and TP were significantly lower in female patients with long COVID who experienced palpitations. This suggests potential changes in autonomic nervous system modulation in patients with long COVID, with a possible decrease in vagal tone. However, since there were no significant changes in LF/HF, we were unable to determine whether vagal regulation was predominant. In conclusion, our study suggests that autonomic dysfunction may contribute to palpitation symptoms in female patients with long COVID.

Dong Y et al. analyzed the HRV of 4,754 outpatients who experienced palpitations (24). The study revealed that 67.7% of these patients had premature ventricular contraction, which was associated with a notable decrease in the HRV time-domain parameter. Their findings suggest that HRV testing can be effectively conducted in individuals with palpitations. The study conducted by Noureldin et al. demonstrated a decrease in SDNN and rMSSD following SARS-CoV-2 infection (25). This decrease was more pronounced in patients who reported experiencing palpitations. Aranyó et al. discovered that post-COVID-19 patients with inappropriate tachycardia had lower levels of SDNN, VLF, LF, and HF compared to the asymptomatic group and healthy controls (26). These results align with the HRV portion of our study. However, Karakayali et al. conducted a study on autonomic dysfunction in post-COVID-19 outpatients and found no significant differences between asymptomatic and asymptomatic patients concerning most HRV parameters, except for PNN50, VLF, and HRVIndex (27). This finding partially contradicts our study, and several factors could contribute to this disparity. For instance, our study subjects consisted exclusively of females, whereas their study subjects included only 50% females. Additionally, variations in the timing of HRV acquisition or other factors may have contributed to these discrepancies. Nonetheless, Karakayali's results showed that HRVIndex was an independent predictor of palpitations and chest pain symptoms in patients after COVID, which is consistent with the last result of this study. In contrast, our study utilized the presence or absence of palpitation symptoms in long COVID patients as a basis for grouping, providing a better illustration of the relationship between palpitation symptoms and HRV in long COVID.

The COMPASS-31 is increasingly being used to screen patients with autonomic dysfunction in the context of long COVID (28, 29). An observational study found that 61.1% of patients with long COVID had a COMPASS-31 score higher than 13.25, which aligns with the findings of the present study where 75% of patients in Group A had a score higher than 13.00 (30). Autonomic dysfunction was observed in 61.1% of patients with long COVID in this study. The higher COMPASS-31 score in Group A, compared to the other two groups, supports the association between palpitation symptoms in female patients with long COVID and autonomic disorders.

The present study found that a percentage of patients with long COVID experienced a decline in their quality of life compared to their pre-infection state with SARS-CoV-2. Specifically, 65.71% of Group A and 41.13% of Group B reported such deterioration. Carfì A et al. conducted a study and showed that 44.1% of patients experienced a decrease in their quality of life after being infected with SARS-CoV-2 (20). Shir LL et al. assessed the quality of life of post-COVID-19 patients across five dimensions (mobility, self-care, daily activities, ache/discomfort, and anxiety/depression) (31). Their results indicated that 48.5% of patients faced quality-of-life issues in at least one of these dimensions. Previous studies have shown that lower quality of life scores are more commonly observed in female populations with persistent symptoms, comorbidities, living alone, and high levels of stress following SARS-CoV-2 infection (32). Another case report revealed that 75% of patients with long COVID had postural tachycardia syndrome, with 70% of them being females (33). These aforementioned studies provide further empirical evidence to support the findings of the present study.

Autonomic dysfunction or dysautonomia is a significant consequence of long COVID, including postural orthostatic tachycardia syndrome (POTS), inappropriate sinus tachycardia (IST), neurocardiogenic syncope (NCS), orthostatic hypotension (OH), and other autonomic disorders (34). Multiple mechanisms are currently believed to contribute to autonomic dysfunction in long COVID. These mechanisms include direct viral injury, mitochondrial dysfunction, sympathetic storm, brainstem dysfunction, oxidative stress, and autoimmunity (6, 35–38). The pathophysiology of direct viral injury involves persistent viral infection triggering a hyperinflammatory state and cellular damage, which ultimately leads to neuronal apoptosis and affects neurological function (39). A study has shown that pro-inflammatory factors such as IL 1-β, IL-6, IL-13, IL-17 A, and TNF *α* are consistently upregulated 7–11 months after SARS-CoV-2 infection (40). Inflammation and hypoxia act as mediators of sympathetic overactivation, while sympathetic overactivation triggers the release of pro-inflammatory cytokines and causes organ damage, thereby exacerbating this vicious cycle (37). Furthermore, studies have found that long COVID is associated with mitochondrial dysfunction, characterized by loss of membrane potential, metabolic dysfunction, redox imbalance, and mitochondrial autophagy (36, 41, 42). Additionally, it has been observed that patients with long COVID can produce more than 20 specific autoantibodies that interfere with neuronal and vascular activity, leading to autonomic dysfunction (43).

According to a study conducted by Blitshteyn et al, 42 patients with post-COVID-19 dysautonomia were observed to have persistent symptoms at one year despite treatment (44). Hence, it is crucial to promptly identify and provide long-term monitoring for patients with long COVID autonomic dysfunction.

## Strengths and limitations

This study is the first to compare long COVID female patients with palpitations, long COVID female patients without palpitations but with other symptoms, and asymptomatic females with a history of COVID-19 using 5 min HRV analysis and the COMPASS-31 scale. The results may contribute valuable data and guidance for future studies.

The single-center study with a small sample size is the main limitation of this study. Additionally, the 5 min HRV was used for initial screening. Furthermore, it is worth mentioning that this study focused on female patients, and although HRV testing was scheduled to be performed within one week after the end of menstruation, there could still be a potential influence of the menstrual cycle on HRV.

## Conclusions

This study revealed that the symptoms of palpitations in female patients with long COVID were linked to HRV parameters. Autonomic dysfunction may be associated with the occurrence of palpitation symptoms in long COVID. Future large-sample follow-up studies are needed to further validate the long-term changes in autonomic function of long COVID in order to better prevent and treat palpitation symptoms or other symptoms in patients with long COVID.

## Data Availability

The original contributions presented in the study are included in the article/Supplementary Material, further inquiries can be directed to the corresponding author.
